# Ampholytic ion-exchange magnetic beads: a promising tool for selecting short fragments in circulating cell-free DNA analysis

**DOI:** 10.3389/fonc.2024.1397680

**Published:** 2024-05-08

**Authors:** Gan He, Weixuan Wang, Yongxia Zhou, Guowei Zhao, Juan Liao

**Affiliations:** ^1^ Gastrointestinal Surgery, Yongchuan Hospital of Chongqing Medical University, Chongqing, China; ^2^ Institute of Life Sciences, Chongqing Medical University, Chongqing, China; ^3^ Department of Radiology, Yongchuan Hospital of Chongqing Medical University, Chongqing, China; ^4^ Center Laboratory, Yongchuan Hospital of Chongqing Medical University, Chongqing, China; ^5^ Chongqing Geriatric Disease Clinical Research Center, Chongqing, China

**Keywords:** ampholytic ion exchange, cell-free DNA, liquid biopsy, magnetic bead, magnetic submicron particle, silane, zwitterion

## Abstract

**Objective:**

For liquid biopsy of cancer, the extraction of circulating cell-free DNA (cfDNA) from plasma is required. We evaluated the efficacy of use of magnetic submicron particles coated with abundant small zwitterions (MSP-ZEWBs) for extracting short fragments of cfDNA.

**Methods:**

We developed and optimized an MSP-ZEWB-based cfDNA extraction method using ampholytic ion-exchange materials and compared its results with those using a control kit. We measured the cfDNA concentration by quantitative polymerase-chain-reaction and using the Qubit method and analyzed cfDNA fragmentation patterns using a bioanalyzer.

**Results:**

The fragment size of cfDNA isolated from glycine hydrochloric acid at a pH of 2.2 exhibited a better alignment with the DNA marker. The highest DNA intensity was observed at the final concentration of 0.8% polyethylene glycol 8000. The intensity of cfDNA decreased significantly when isolated from plasma with DNA marker using MSP-ZEWBs with an adsorption buffer containing guanidine hydrochloride or isothiocyanoguanidine. All fragments were successfully extracted using MSP-ZEWBs from both plasma and phosphate-buffered saline. Notably, the intensity of short cfDNA fragments isolated using MSP-ZEWBs remained consistent for recovery of long DNA fragments. indicating a potential selective of small fragments.

**Conclusion:**

The extraction of plasma cfDNA with MSP-ZEWBs requires no protein denaturation, shows resistance to cells remaining in plasma, and demonstrates higher overall efficiency and better reproducibility than other extraction methods. Use of MSP-ZEWBs may greatly enhance liquid biopsy of cancers through the analysis of plasma cfDNA in clinical practice.

## 1 Introduction

Cancer is fundamentally a disease caused by genetic mutations that disrupt the delicate homeostasis of cellular functions (1). For liquid biopsy of cancers, circulating cell-free DNA (cfDNA) has been explored as a potential biomarker for cancer screening and monitoring (2–4). Typically, biomarker isolation is one of three important steps in liquid biopsy workflow (5). In terms of size analysis, cfDNA mostly contained fragments less than 1000 base pairs (bp) (6). These cfDNA fragments are derived from nuclease-processed nucleosome arrays which consist of 147 bp of DNA wrapped around a histone octamer core with approximately 20-40 bp of unbound linker DNA interspersed (7, 8). Short cfDNA fragments(<150 bp) contain a larger fraction of mutated fragments than the total cfDNA pool (9). However, commercial cfDNA extraction kits shown significant variability with respect to recovery, yield reproducibility, and fragment sizes for the isolation of cfDNA (10–12). A lower isolation efficiency of short fragments were reported in part of commercial kits (12). The sensitivity and specificity of cfDNA may be enhanced by selection of a cfDNA extraction method that favors the recovery of short frgments (11). The cfDNA extraction kits have a profound effect on cfDNA yield, where efficient recovery is desired for downstream analyses (13). These low quantity and quality of cfDNA are challenging for downstream assays such as whole genome sequencing and SNP genotyping (14). Therefore, cfDNA yield and stability is required for liquid biopsy of cancers through the analyses of mutations and methylation profiling methods achieving this remains a technical challenge (5).

For the extraction of plasma cfDNA, phase isolation, silane membrane filtration, and silane membrane adsorption can be used (2, 15). In comparison with that of phase separation, adsorption or filtration with silane membranes is more advantageous for extracting plasma cfDNA, as it has higher operation efficiency and does not require use of toxic reagents (16–18). In practice, silane membrane filtration is facilitated by centrifugation with spin columns bearing silane surfaces, whereas silane membrane adsorption is usually realized through magnetic separation with magnetic submicron particles (MSPs) bearing silane surfaces. Silane membrane adsorption of cfDNA *via* magnetic separation is more readily automated and more advantageous than other processes. On silane membranes, however, heavy nonspecific adsorption of proteins interfere with the subsequent analyses of plasma cfDNA. Protein denaturation prevents such interference but breaks leftover cells and releases intracellular DNA, complicating the extraction/analyses of plasma cfDNA. Another challenge is that different approaches leave an abundance of leftover cells in plasma and thus different yields of plasma cfDNA. To overcome these challenges, two-step centrifugation of venous blood to remove white blood cells as completely as possible is required (16–18) but currently exhibits insufficient efficacy and is very time consuming. To meet the demands of high capacity conditions, automated cfDNA extraction methods may be ideal (11).

To promote liquid biopsy of cancers, new biomaterials and methods resistant to blood cells remaining in plasma are thus highly desired for rapid extraction of plasma cfDNA. Application of commercial beads has typically been limited to the target DNA fragments. A recent report described a solid-phase reversible immobilization (SPRI) beads-based DNA purification strategy that provides flexibility and cost effectiveness (19). A liquid-phase extraction method of cfDNA based on aqueous two-phase systems indicated higher cfDNA recovery (20). New ampholytic ion-exchange materials have been patented (21–24) and engineered on MSPs coated with abundant small zwitterions to yield a series of subtypes denoted as MSP-ZEWBs (Bolanying, Chongqing, China). Preliminary studies found that MSP-ZEWBs readily adsorbed purified plasmids at a pH of approximately 4.0 in the absence of proteins and that the adsorbed plasmids were easily eluted at a pH of approximately 8.9. Notably, MSP-ZEWBs showed negligible nonspecific adsorptions of proteins (25), indicating that the adsorption of plasma cfDNA with MSP-ZEWBs at a pH of approximately 4.0 may need no protein denaturation and be resistant to cells remaining in plasma. For comparison of plasma cfDNA yields based on the quantification of a DNA sequence in plasma cfDNA *via* quantitative polymerase-chain-reaction (qPCR), a coding fragment in an exon of the Alu gene found in common blood cells is a favorable model to examined the interference of remaining blood cells in plasmas (26, 27). In our study, qPCR of a coding fragment of the Alu gene revealed that the extraction of plasma cfDNA with MSP-ZEWBs required no denaturation of proteins, prevented cells from remaining in the plasma, and was sufficiently absorbed to promote liquid biopsy of cancers.

## 2 Materials and methods

### 2.1 Materials and chemicals

A MagMAX Cell-Free DNA Isolation Kit, which uses magnetic silane for cfDNA adsorption, was provided by Thermo Fisher Scientific (Waltham, MA, USA). A Protease K and Quant-iT dsDNA Assay kit were purchased from Invitrogen (Waltham, MA, USA). TB Green Premix Ex TaqII (Tli RNase H Plus) was provided by TaKaRa (Tokyo, Japan). Other chemicals used were analytical reagents. Plasma was prepared using Heal Force Neofuge 13R centrifuges under stated centrifugation forces (Heal Force, Shanghai, China). Quantity and quality were assessed with an Agilent 4150 TapeStation system with DNA1000 Screen Tape analysis (Agilent, Santa Clara, CA, USA). All extractions were performed using a Thermo Fisher Scientific KingFisher Duo Prime Purification System. A BioRad CFX96 real-time system was used as the purification system in the work (BioRad, Hercules, CA, USA).

### 2.2 Preparation of MSP-ZEWBs

MSP-ZEWBs bearing an apparent isoelectric point of approximately 6.4 after careful optimization of the preparation process were prepared according to a procedure described previously (21). In brief, naked MSPs bearing moderately hydrophilic surfaces and flexible carboxyl groups were prepared according to a previously reported procedure (28). The naked MSPs were then coated with abundant small zwitterions and flexible carboxyl groups according to a patented approach (23). Modification of the coated MSPs with lysine alone yielded MSP-ZEWBs (21). Commercialized MSP-ZEWBs products have the isoelectric points described above and have already been manufactured at 200 g/batch, which is suitable for industrial applications.

### 2.3 Patient selection

Venous blood samples were collected and stored in the central laboratory of Yongchuan Hospital of the Chongqing Medical University. In total, 10 healthy volunteers and 29 colon cancer patients were enrolled in the study. To optimize cfDNA isolation of MSP-ZEWBs and minimize the contribution of biological variation, we obtained a pooled plasma with 10 healthy volunteers. The plasma pool of 5 colon cancer patients was created to assess the reproducibility of MSP-ZEWBs and influence of denaturation reagents. Furthermore, a comparison of the performance of MSP-ZEWBs and the commercial extraction kit was conducted using a 24 colon cancer patients. The work was approved by the ethics committee and written informed consent was obtained from each participant.

### 2.4 Extraction of cfDNA from plasma

Peripheral blood was collected in K_2_-EDTA vacutainers and prepared within two hours through centrifugation under stated conditions at 4°C to obtain plasma, whose aliquots were stored at −20°C before use. For extraction of cfDNA with MSP-ZEWBs, 300 μL of plasma and 10 to 40 μL of proteinase K (20 g/L) were mixed before the MSP-ZEWBs were added at an indicated quantity in 50 to 1200 μL of an adsorption buffer, mixed, and incubated at room temperature for adsorption within 2 to 10 min. The MSP-ZEWBs together with the adsorbed cfDNA were recovered by magnetic separation in 2.0 min. After the supernatant was carefully removed with a vacuum pump, the MSP-ZEWBs were washed with 60% ethanol and wash buffer. Finally, the adsorbed cfDNA was eluted with 40 μL of 25 mmol L^-1^ Tris-HCl buffer at pH 8.9 within 2 to 10 min at room temperature. The removal of the MSP-ZEWBs yielded cfDNA in the eluent that was directly analyzed by qPCR or stored at −80°C.

### 2.5 Quantification of Alu gene copy by qPCR

The purity of cfDNA was first verified with a NanoDrop 2000 spectrophotometer (Thermo Scientific, Waltham, MA, USA) based on a 260 to 280 nm ratio of the absorbance of cfDNA. The concentration of cfDNA was measured using a Quant-iT dsDNA Assay Kit (Thermo Scientific). To quantify the cfDNA yield, a fragment of 247 and 115 bp of the Alu gene sequence was amplified with a pair of primers following the standard protocol with the BioRad CFX 96 real-time system (26, 27, 29). For amplification with the Bio-Rad system, following protocol was used: 95 C for 30 s, 39 cycles at 95°C for 30 s (denaturation), 59°C for 30 s (annealing), and 72°C for 1 min (extension) including the plate read. A melt curve from 65 to 95°C with 0.5°C increments for 5 s followed each run.

### 2.6 Ladder DNA purification

Ladder DNA (GeneRuler, Thermo Fisher Scientific) was used to simulate cell-free DNA, which is known to contain short (under 1000 bp) fragments. After 300 µL of plasma was added to 5 µL of ladder DNA, each purification method used magnetic beads to recover DNA. The volume of beads used was determined according to the manufacturer’s recommendations, and the beads were eluted into 50 µL of TE or elution buffer. A sensitive PCR-based quantification method was not appropriate for ladder DNA purification because of the absence of primer-binding sites and continuous template. The purified DNA was quantified again with 4150. Purification kits designed for cfDNA purification from plasma using MSP-ZEWBs and the MagMAX Cell-Free DNA Isolation Kit (Thermo Fisher Scientific) used as the control were trialed. Ladder DNA added to plasma was purified using the manufacturer’s protocol for each purification method.

### 2.7 Effects of protein denaturation on cfDNA extraction

Guanidine hydrochloride was used in the adsorption systems containing 1200 μL or 300 μL of plasma, 20 μL of proteinase K, and 2.0 mg of MSP-ZEWs in 20 mM of sodium acetate at pH 4.0 to test the effects of protein denaturation. After adsorption for 5 or 10 min, the MSP-ZEWs were recovered and washed once with 800 μL of 60% ethanol in 20 mM of sodium acetate at a pH of 4.0 and twice with 800 μL of 20 mM sodium acetate at a pH of approximately 4.0. Finally, cfDNA was eluted in 40 μL of 25 mM Tris-HCl at pH 8.9, with the use of 2.0 mg MSP requiring no less than 35 μL of the elution buffer). To make artificial plasma containing the remaining blood cells, 0.5 mL of lymphocyte cells was separated from 15 mL of blood using the standard protocol, and the number of cells was counted. Plasma for the indicated concentrations of lymphocyte cells was prepared to test the interference of remaining cells with the extraction of cfDNA by MSP-ZEWs.

### 2.8 Data analysis

Statistical analyses were performed with SPSS version 26.0 (IBM, Armonk, NY, USA). Differences were examined by paired-samples *t* tests and the results reported as the mean ± standard deviation(SD). The concentration at ~180 bp of cfDNA was described by the median and interquartile range and analyzed with Wilcoxon signed Ranks Test. The results of error analysis were reported as the SD of the variance; *P* < 0.05 indicated statistical significance.

## 3 Results

### 3.1 Theoretical considerations in use of MSP-ZEWBs

The MSP-ZEWBs had apparent isoelectric points at pH > 5.5 (21) and thus positive net charges at a pH of approximately 4.0. DNA continues to carry multiple negative charges at this pH, thus being easily adsorbed on MSP-ZEWBs. The isoelectric point of calves blood plasma proteins was ranging from 4.0 to 7.0 (30). The protein peak at pI 5.99 was detected in human serum sample, and minimum proteins with pI less than 4.0 was detected after the marker of lucose oxidase (pI 4.2) (31). Therefore, few proteins have isoelectric points lower than a pH of 4.0 and thus negative charges at a pH of approximately 4.0. At a pH of approximately 4.0, negligible adsorption of plasma proteins occurs on MSP-ZEWBs *via* electrostatic attraction because of electrostatic repulsion. However, coating of particles with abundant small zwitterions putatively decreases non-electrostatic adsorption of common proteins. As the tested MSP-ZEWBs were made of ampholytic groups on an MSP coated with abundant small zwitterions (21–23), there was negligible non-electrostatic interaction between the MSP-ZEWBs and common proteins. As unwanted adsorption of plasma protein should be negligible at pH of approximately 4.0, the extraction of plasma cfDNA after optimization may need no protein denaturation.

### 3.2 Determination of pH of adsorption buffers

**Figure 1:**
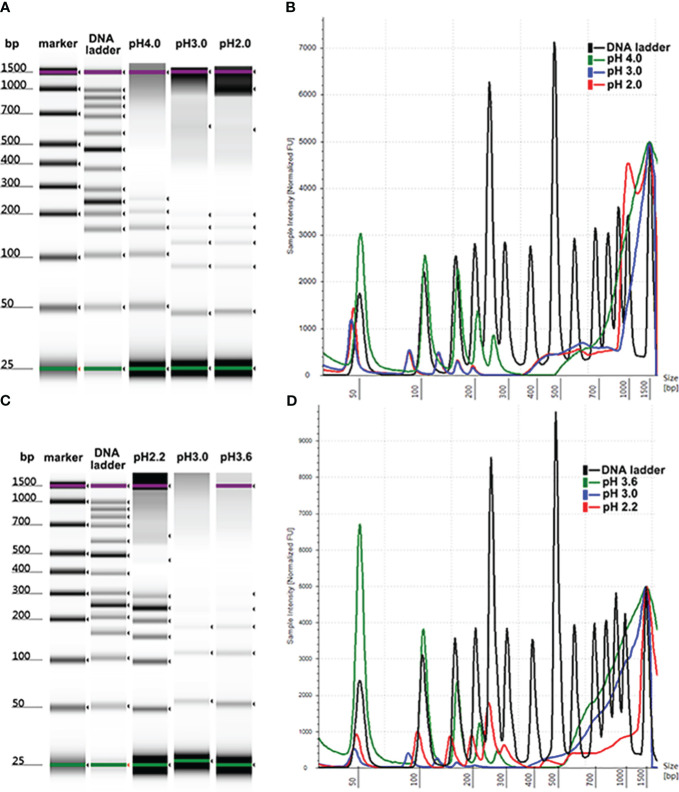
Size profiles of DNA ladder isolated by MSP-ZEWB with different adsorption buffers. **(A)** Gel image of DNA ladder recovered by MSP-ZEWBs with adsorption buffer from sodium acetate at a pH of 2.0–4.0. **(B)** The DNA ladder recovery was highest in the adsorption buffer at a pH of approximately 4.0. **(C)** Gel image of DNA ladder recovered by MSP-ZEWBs with adsorption buffer from glycine hydrochloric acid at a pH of 2.2–3.6. **(D)** The fragment size of DNA ladder isolated from glycine hydrochloric acid at a pH of 2.2 exhibited the best alignment with the DNA ladder.

To optimize the conditions for cfDNA extraction using MSP-ZEWBs, plasma was prepared by two-step centrifugation of venous blood at 1,600 ×*g* for 10 min before the supernatant was centrifugated at 16,000 ×*g* for 10 min. As magnetic submicron particles coated with abundant small zwitterions, MSP-ZEWBs may exhibit a different extraction mechanism compared with magnetic beads coated with carboxyl or hydroxyl groups. Thus, prior to cfDNA extraction, the adsorption conditions were optimized. According to the adsorption mechanism of DNA on MSP-ZEWBs, both ionic strength and pH for adsorption needed optimization for the highest yield of plasma cfDNA considering the complex composition of human plasma. The adsorption of cfDNA on MSP-ZEWBs more easily occurs at a lower ionic strength and lower pH. Due to its strong enzymatic activity, use of 20 μL of proteinase K may be sufficient for 300 μL of plasma. After the pH of sodium acetate as adsorption buffer was evaluated, 20 mM of sodium acetate at a pH from 2.0 to 4.0 was used as the adsorption buffer (**Figures 1A, B**). At an indicated volume, the sodium acetate was mixed with 300 μL of plasma and 20 μL of proteinase K in the adsorption system. DNA ladder recovery was highest in an adsorption buffer at a pH of approximately 4.0 because of the effective action of proteinase K in inactivating enzymes by hydrolyzing nucleic acids required at a pH > 4.0.

The molecular structure of DNA was altered by dehydration in the presence of polyethylene glycol (PEG) and sodium chloride (NaCl) or magnesium chloride (MgCl_2_). Consequently, these negatively charged phosphate groups of DNA were exposed and binded by negatively charged carboxyl groups on the surface of the magnetic beads. The establishment of the ion bridge facilitated by dissociated salt ions in the adsorption buffer and the presence of carboxyl groups of magnetic beads enhanced the selective adsorption of cfDNA in the isolation mechanism of carboxyl-coated magnetic beads. To investigate the mechanism of MSP-ZEWBs, glycine hydrochloric acid without salt ions was selected as the adsorption buffer within a pH range of 2.0–3.6 (**Figures 1C, D**). The highest intensity of DNA ladder was observed in the adsorption buffer at a pH of approximately 3.6. However, the fragment size of DNA ladder isolated from glycine hydrochloric acid at a pH of 2.2 exhibited better alignment with the DNA marker.

The formation of the ion bridge occurred between the positively charged zwitterions of the MSP-ZEWBs and the negatively charged phosphate groups, promoting the adsorption of DNA onto the magnetic bead surface. In theory, as the pH decreases, the magnetic beads exhibit an increased positive charge, thereby enhancing their adsorption capacity. The intensity of DNA isolated from glycine hydrochloric acid at a pH of 2.2 was lower than that at 3.6, which was attributed to the efficient activity of proteinase K in deactivating enzymes that hydrolyze nucleic acids required at a pH > 4.0. Otherwise, the intensity of DNA ladder isolated from glycine hydrochloric acid at a pH of 2.2 was lower than that from sodium acetate, possibly because of incomplete exposure of phosphate groups due to a lack of PEG and NaCl in the adsorption buffer.

### 3.3 Determination of PEG concentration

The reduced intensity of DNA isolated by MSP-ZEWBs using glycine hydrochloric acid as the binding buffer may be attributed to a lower quantity of negatively charged phosphate groups binding with the positively charged zwitterions. The role of PEG at a specific molecular weight was to dehydrate the DNA and alter the molecular conformation, allowing more negatively charged phosphate groups to be exposed. By enhancing the interaction with the positively charged zwitterions of the MSP-ZEWBs, this exposure optimized the efficiency of DNA extraction. This interaction enhanced the viscosity of the system, facilitating the sustained suspension and functionality of the magnetic beads. By preventing easy settling and promoting increased collision and rejection of the magnetic beads in their spatial position, this sustained suspension and functionality enhanced the aggregation efficiency and effectiveness of nucleic acid and magnetic beads. Additionally, PEG exhibited compatibility with proteins and effectively eliminated proteins from the sample.

**Figure 2:**
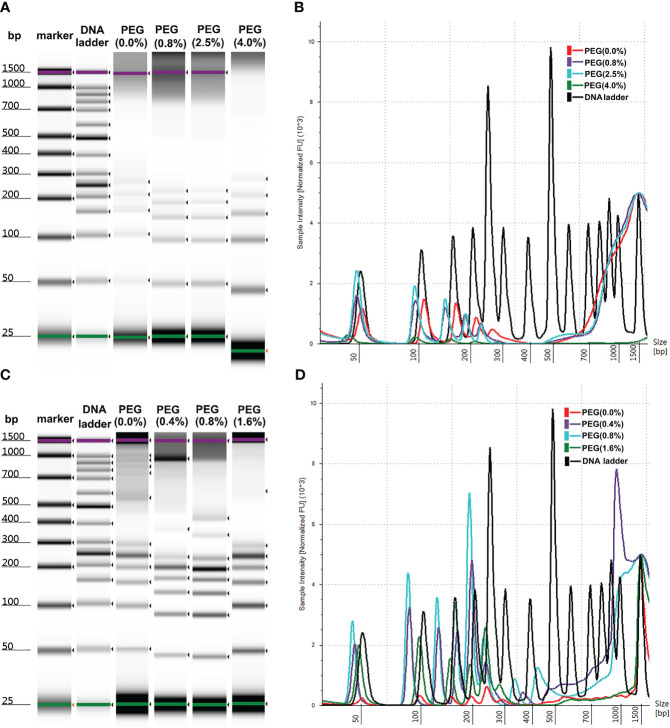
Size profiles of DNA **ladder** isolated by MSP-ZEWB with adsorption buffer containing PEG 8000. **(A)** Gel image of DNA ladder isolated by MSP-ZEWBs with final concentrations of PEG 8000 ranging from 0% to 4.0% in sodium acetate. **(B)** No statistically significant increase was observed. **(C)** Gel image of DNA ladder isolated by MSP-ZEWBs with final concentration of PEG 8000 ranging from 0% to 1.6% in glycine hydrochloric acid. **(D)** The highest intensity of DNA ladder was observed at a final concentration of 0.8% PEG 8000.

As shown in **Figure 2**, no statistically significant increase was observed in the intensity of DNA ladder isolated by MSP-ZEWBs with sodium acetate at a pH of 4.0. The yield of DNA ladder isolated from plasma using glycine hydrochloric acid as a binding buffer containing PEG 8000 was > 10 times greater than that of DNA ladder extracted with glycine hydrochloric acid alone, as depicted. However, the efficacy of DNA extraction was not directly proportional to the amount of PEG 8000 used. As the highest intensity of DNA ladder was observed at a final concentration of 0.8% PEG 8000, this proportion was used in the binding solution for cfDNA extraction from 300 μL of plasma unless otherwise specified.

### 3.4 Determination of denaturation reagents

**Figure 3:**
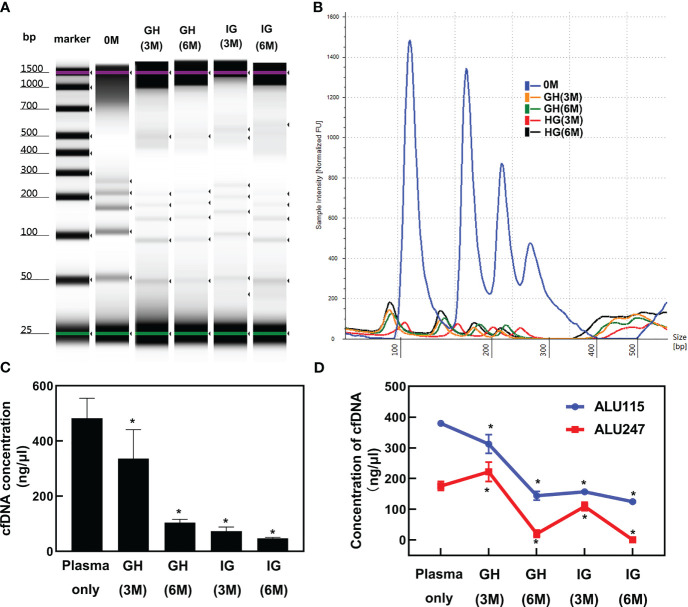
Effect of denaturation reagents on DNA isolated by MSP-ZEWBs. **(A)** Gel image of DNA ladder extracted by MSP-ZEWBs with adsorption buffer containing guanidine hydrochloride(GH) or isothiocyanoguanidine(IG). **(B)** The intensity of DNA ladder sharply decreased when isolated from plasma with DNA markers using MSP-ZEWBs with the adsorption buffer containing denaturation reagents. **(C)** The quant-iT method was used to analyze the concentration of cfDNA isolated from plasma only. A significant decrease in cfDNA concentration was observed in plasma isolated by MSP-ZEWBs using the adsorption buffer containing denaturation reagents compared with that using the adsorption buffers without denaturation reagents (*P* < 0.05). **(D)** qPCR was employed to detect the concentration of cfDNA isolated from plasma with amplicons Alu 115 and Alu 247. *Significant difference between adsorption buffer without denaturation reagents and with denaturation reagents at *P* < 0.05. OM: DNA ladder in plasma with no denaturation reagents. GH(3M): DNA ladder in plasma with final concentration at 3 mol/L of guanidine hydrochloride. GH(6M): DNA ladder in plasma with final concentration at 6 mol/L of guanidine hydrochloride. IG(3M): DNA ladder in plasma with final concentration at 3 mol/L of isothiocyanoguanidine. IG(6M): DNA ladder in plasma with final concentration at 6 mol/L of isothiocyanoguanidine.

Commercial reagent kits were employed for the extraction of plasma cfDNA with magnetic silanes to terminate the non-specific adsorption of proteins. The denaturation reagents that were used lysed the cells and introduced genomic contamination. To test the effects of protein denaturation on the yields of plasma cfDNA with MSP-ZEWBs, guanidine hydrochloride and isothiocyanoguanidine were employed, as the adsorption of purified plasmids on MSP-ZEWBs are susceptible to common detergents but reasonably resistant to monovalent anions (21, 25). The intensity of DNA ladder was decreased sharply in presence of denaturations reagent (**Figure 3A**). The copy numbers of the Alu gene in cfDNA from plasma extracted with MSP-ZEWBs under the optimized conditions were compared with those of cfDNA from plasma extracted with adsorption buffer containing denaturation reagents (**Figure 3B**). The concentration of cfDNA was significantly lower in the former (*P* < 0.05) (**Figure 3C**).

When the concentration of cfDNA isolated from plasma was detected by qPCR with amplicons Alu 115 and Alu 247 (**Figure 3D**). the concentration of cfDNA isolated using MSP-ZEWBs with an adsorption buffer containing isothiocyanoguanidine or guanidine hydrocholoride was lower than that with an adsorption buffer without denaturation reagents. The nucleic acid was adsorbed with electrostatic attraction by an ion-exchange magnetic bead not selective for anions, with the efficiency of magnetic-bead extraction of nucleic acid influenced by competitive binding of other anions in the sample, neutralization of nucleic acid charge by polyvalent metal ions, and other factors. The MSP-ZEWB surface possesses a zwitterion pair modification layer, resulting in negligible non-specific protein adsorption and thereby eliminating the need for denatured protein during nucleic acid extraction. In fact, guanidine hydrochloride and isothiocyanoguanidine for effective denaturation of proteins reduced the yields of the cfDNA in plasma cfDNA extracted with the MSP-ZEWBs.

In the presence of guanidine hydrochloride and isothiocyanoguanidine, the minimum yields of cfDNA in plasma indicated the competitive adsorption of concentrated chloride anions against cfDNA. In addition, the reduced yields of cfDNA in plasma following protein denaturation may have partially been caused by reduced adsorption of cfDNA due to the release of lipids from denatured lipoproteins. To further mitigate the potential effects of lipids released from lipoproteins on denaturation, the wash process was optimized, but using wash buffers did not improve the yields of cfDNA in plasma with guanidine hydrochloride or isothiocyanoguanidine.

### 3.5 Determination of magnetic bead concentration

**Figure 4:**
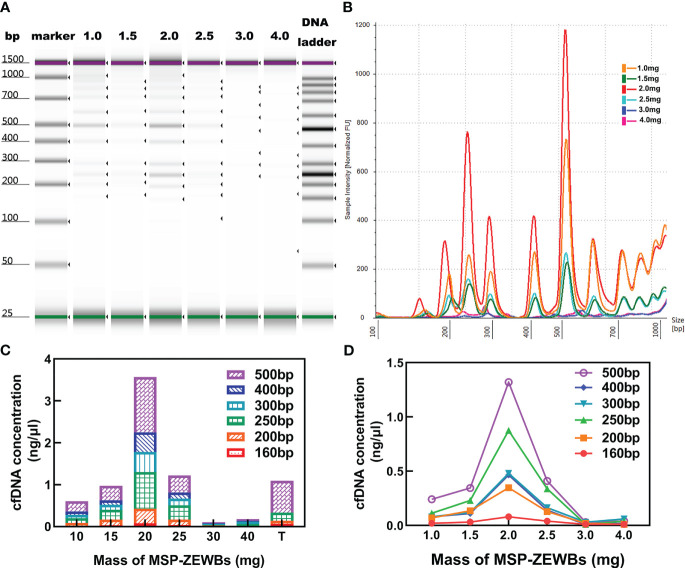
Effects of MSP-ZEWBs and plasma quantities on yields of DNA ladder. **(A)** Gel image of DNA ladder extracted by MSP-ZEWBs from 1.0mg to 4.0mg. **(B)** Ratio of mass weight of MSP-ZEWBs to extracted DNA ladder from plasma. **(C)** Concentration of DNA ladder in different fragment sizes. **(D)** cfDNA concentration increased in the quantity of MSP-ZEWBs up to 2.0 mg.

The concentration of cfDNA was affected by the quantity of MSP-ZEWBs, as evidenced by the continued increase of cfDNA intensity with increases in the quantity of MSP-ZEWBs up to 2.0 mg (**Figures 4A, B**). However, the intensity diminishes notably with quantities of MSP-ZEWBs beyond this threshold, indicating a specific requirement for magnetic-bead dosage. Notably, within a defined concentration range, the concentration of DNA ladder exhibited a direct relationship with magnetic-bead dosage. This relationship was further evidenced by the varying extraction concentrations of the different fragment sizes (**Figures 4C, D**). The yields of DNA ladder in 300 μL of plasma increased up to MSP-ZEWBs quantities of 2.0 mg. Clearly, the use of only 2.0 mg of MSP-ZEWBs is preferred because of the high cost of patented MSP-ZEWs.

Altogether, the adsorption of plasma cfDNA isolated by 2.0 mg of MSP-ZEWBs in a glycine hydrochloride buffer containing 0.8% PEG 8000. After adsorption at 25°C for 10 min, the recovered MSP-ZEWBs were washed with glycine hydrochloride buffer and 60% ethanol two times. Elution with 40 μL of 25 mmol L^-1^ Tris-HCl at pH 8.9 for 4.0 min yielded plasma cfDNA in the eluent.

### 3.6 Comparison of cfDNA extraction kits

**Figure 5:**
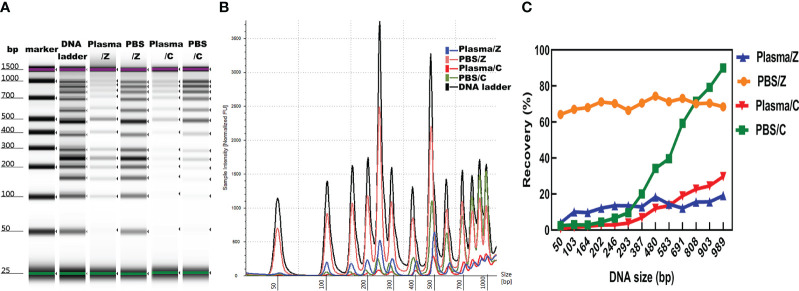
Effect of solution on DNA ladder isolation. **(A)** Gel image of DNA ladder isolated by MSP-ZEWBs and by the control kit from plasma or PBS. **(B)** Bioanalyzer traces comparing the intensity of DNA ladder. **(C)** Mean recovery percentage of different fragments of DNA ladder isolated by MSP-ZEWBs and by the control kit from plasma or PBS. Z: DNA ladder was isolated by MSP-ZEWBs. C: DNA ladder was isolated by the control kit.

The concentration, distribution, and recovery of DNA ladder isolated by MSP-ZEWBs were calculated and subsequently compared with those of DNA ladder extracted with a control kit both in phosphate-buffered saline (PBS) and plasma. A bioanalyzer was used to analyze the size profiles of DNA ladder isolated by MSP-ZEWBs and the control kit from plasma or PBS. The intensity of DNA ladder isolated by MSP-ZEWBs and the control kit was higher in PBS than that in plasma (**Figure 5A**). The intensity of DNA ladder isolated by MSP-ZEWBs was higher than that by the control kit, suggesting that the extraction efficiency of DNA ladder may be enhanced using ion-exchange magnetic beads under modified conditions as opposed to silane magnetic beads.

The different fragments of DNA ladder were successfully extracted by MSP-ZEWBs from both plasma and PBS. Notably, the intensity of large fragments isolated by the control kit exceeded that of small fragments, indicating a potential loss of small fragments (**Figure 5B**). Calculation of the mean recovery percentage of different fragments revealed that use of MSP-ZEWBs demonstrated superior performance compared with that of the control kit in both PBS and plasma, yielding mean recovery rates of 69.7 and 32.5%, respectively (**Figure 5C**). The recovery rates of MSP-ZEWBs in PBS and plasma were 69.7 and 13.1%, respectively. MSP-ZEWBs performance remained consistent for recovery of DNA ladder. The recovery rate of a control kit was 32.5% in PBS and 10.9% in plasma, indicating higher recovery with larger fragments of DNA ladder. The lower recovery rate in plasma than PBS can be attributed to the composition of plasma, a result consistent with that reported for recovery of DNA fragments (11, 32).

### 3.7 Application of MSP-ZEWBs to the extraction of plasma cfDNA

Reproducibility of cfDNA extraction using MSP-ZEWBs from plasma was assessed With the same spiked plasma, the values of Ct of amplicons Alu 115 and Alu 247 with MSP-ZEWBs were determined to be 16.3 ± 0.2 (n =5) and 15.4 ± 0.6 (n = 5), respectively. The coefficients of variation for these values were determined to be 1% and 4%, respectively, both falling below the accepted threshold of 10%. These results indicated a high level of consistency in the adsorption of cfDNA onto MSP-ZEWBs.

To further validate the utility of MSP-ZEWBs, the concentration of cfDNA was evaluated using the quant-iT method, qPCR, and bioanalyzer. The results indicated no significant difference in the mass concentration of cfDNA between the MSP-ZEWBs and control group (MSP-ZEWBs: (75.2 ± 0.9) ng/ul; control group: (75.5 ± 1.2) ng/ul) as determined by the quant-iT method (**Table 1**). The MSP-ZEWBs group exhibited a higher level of short fragments compared to the control group (MSP-ZEWBs: (4.0 ± 0.5) ng/ml; control group: (3.8 ± 0.7) ng/ul, *P*=0.002). Additionally, the MSP-ZEWBs group showed a lower level of long fragments of cfDNA amplified by 247 bp Alu amplicons in comparison to the control group, with no significant difference observed between the two groups (MSP-ZEWBs: (16.0 ± 7.4) ng/ml; control group: (16.9 ± 8.9) ng/ul, *P*=0.484). There was also no significant difference in concentration at ~180 bp, as measured by bioanalyzer, between the MSP-ZEWBs and control groups (MSP-ZEWBs: 28.5(12.3-46.1)ng/ml; control group: 19.8(9.2-34.9) ng/ul, *P*=0.074). Hence, the concentration and fragment distribution of cfDNA isolated by MSP-ZEWBs exhibited consistency with the control group, supporting the applicability of MSP-ZEWBs.

**Table 1:** concentration of cfDNA with MSP-ZEWB and a control group.

	MSP-ZEWBs (n=24)	Control group (n=24)	*t*	*P*
Concentration(ng/ul) in quant-iT method	75.2 ± 0.9	75.5 ± 1.2	2.067	0.050
Alu 115(ng/ml)	4.0 ± 0.5	3.8 ± 0.7	3.483	0.002^*^
Alu 247(ng/ml)	16.0 ± 7.4	16.9 ± 8.9	0.712	0.484
cfDNA integrity	3.9 ± 1.3	4.3 ± 1.5	2.880	0.008^*^
Concentration(ng/ml) at ~180 bp	28.5(12.3-46.1)	19.8(9.2-34.9)	1.784	0.074

*Compare to the control group, the difference was statistically significant (P < 0.05).

## 4 Discussion

Liquid biopsy of cancers through the analysis of plasma cfDNA appears promising, but the best method for performing it remains unclear (2, 15, 33, 34). For the preparation of plasma, one-step centrifugation of approximately 10 min is highly efficient (29, 35–38) but inevitably leads to white blood cells remaining in the plasma. To date, protein denaturation is required for the extraction of plasma cfDNA with traditionally used silane membranes (39), a process that may lead to blood cells remaining in the plasma after extraction of plasma cfDNA. Different methods of plasma preparation yield different quantities of white blood cells remaining in the plasma and thus different levels of interference in the plasma cfDNA extracted after protein denaturation. Such differences may have led to the controversy regarding the best method for liquid biopsy of cancers through the analysis of plasma cfDNA (2, 15, 33, 34). Notably, use of MSP-ZEWBs coated with abundant small zwitterions showed negligible nonspecific adsorption of proteins (23, 25); indeed, the extraction of plasma cfDNA with MSP-ZEWBs required no protein denaturation and was resistant to white blood cells remaining in the plasma.

Use of MSP-ZEWBs demonstrated superior performance compared with use of the control kit in both PBS and plasma. All extractions were performed using the KingFisher Duo Prime Purification System (Thermo Fisher Scientific). Because the quantity of plasma used was 300 μL in both the MSP-ZEWB extraction system and the control kit, use of the control kit was not conducted strictly in accordance with the instructions of the manufacturer, and the results therefore do not represent extraction levels. However, a stronger adsorption of cfDNA through MSP-ZEWBs suggested its potential advantages for the extraction of trace viral nucleic acids. The results of analysis of cfDNA in bodily fluids were also promising for the diagnosis of diseases other than cancer, and the extraction of cfDNA with MSP-ZEWBs avoided potential interference from cells remaining in bodily fluids. Therefore, the extraction of cfDNA with use of MSP-ZEWBs from plasma and other bodily fluids showed significant advantages over the use of magnetic silanes.

The extraction of cfDNA from plasma based on spin-column is currently cumbersome, difficult to automate, time-consuming and costly for handling large number of samples. The Qiagen QIAamp Circulating Nucleic Acid Kit, as the most commonly used for plasma cfDNA, was almost needed 2 hours and cost 25 dollar per sample (40). The control commercial kit in our study cost 14 dollar per sample, while the MSP-ZEWBs just cost 2.5 dollar per sample. In general, about 30 min are required for the total extraction of plasma cfDNA with MSP-ZEWBs automatically. The time for adsorption and elution of plasma cfDNA with MSP-ZEWBs manually was only 9 min, in addition to the approximate 3 min required for washing the recovered MSP-ZEWBs after adsorption. The extraction of plasma cfDNA with MSP-ZEWBs showed the highest overall efficiency to date even when the time saved for one-step centrifugation to prepare plasma was not considered. Therefore, MSP-ZEWBs appear promising for use as adsorbing MSP-ZEWBs for the rapid extraction of trace quantities of DNA in biological samples.

The adsorption of DNA with MSP-ZEWBs at a pH of approximately 4.0 relies on electrostatic attraction (21, 24). This type of interaction supports neither enhancement of the analytical sensitivity of plasma cfDNA nor selection of MSP-ZEWBs among DNA fragments of different lengths and other anions in biological samples. The ratios of copy numbers of approximately 100 bp fragments to approximately 200 bp fragments of some mutated genes in plasma cfDNA are better diagnostic indicators for liquid biopsy of cancers (27, 41–43), indicating that the recovery of short DNA fragments from plasma determines the diagnostic significance of the biopsy results through the analyses of plasma cfDNA. Traditional magnetic silanes yield consistent recovery of DNA fragments of 500 bp and larger but increasingly lower recovery of DNA as fragments decrease in size from 500 bp (44, 45). Notably, for the extraction of DNA fragments from 70 to 500 bp, our preliminary data support consistent recovery with use of MSP-ZEWBs but much lower yields of shorter fragments than longer fragments with magnetic silane. Currently, the ratios of copy numbers of fragments bearing different lengths in plasma cfDNA extracted with MSP-ZEWBs are being compared with those extracted with magnetic silane to test the significance of use of MSP-ZEWBs in the liquid biopsy of cancers.

It is important to note that our study has limitations. A limitation of our study is only 24 colon cancer patients included. Furthermore, only 9 colon cancer patients of 24 volunteers had K-RAS gene mutation in their tumor tissues, with an average mutation rate of less than 20%. Consequently, the mutational experiments between the MSP-ZEWBs and the control extraction kit were not fully completed. The personal gene expression profile was note assessed based on the cfChIP-seq of cfDNA. As a result, the our findings need to be repeated in larger patients and expanded to include other types of cancer. Further additional research is needed, such as cfDNA fragmentomics and methylation analysis to establish robust scientific evidence for MSP-ZEWBs as a promising tool for selecting short fragments in cfDNA.

## 5 Conclusion

In this study, the use of MSP-ZEWBs, ampholytic ion-exchange magnetic beads, showed consistent recovery of short and long DNA fragments with higher overall efficiency and better reproducibility. Further, it was found that the extraction of plasma cfDNA with MSP-ZEWBs required no protein denaturation, showed resistance to cells remaining in plasma. It is a promising tool for selecting short fragments in cfDNA analysis. As such, the use of MSP-ZEWBs may greatly enhance liquid biopsy of cancers through the analysis of plasma cfDNA in clinical practice.

## Data availability statement

The original contributions presented in the study are included in the article/supplementary material. Further inquiries can be directed to the corresponding author.

## Ethics statement

The studies involving humans were approved by Ethics Committee of Yongchuan Hospital of Chongqing Medical University (approval No. 2020KLS022). The studies were conducted in accordance with the local legislation and institutional requirements. The participants provided their written informed consent to participate in this study.

## Author contributions

GH: Writing – review & editing, Investigation, Writing – original draft, Project administration. WW: Validation, Writing – review & editing. YZ: Writing – review & editing. GZ: Data curation, Writing – review & editing, Project administration. JL: Writing – original draft, Project administration, Methodology, Funding acquisition, Formal Analysis, Writing – review & editing.
